# **PROCEEDINGS OF THE 17**^**th**^**ITALIAN CONVENTION OF FFC INVESTIGATORS IN CYSTIC FIBROSIS**: VERONA 14^th^-16^th^ NOVEMBER 2019 Every year, the Italian Cystic Fibrosis Research Foundation (FFC) brings together all its funded researchers from across Italy and beyond, in a Convention where results from FFC projects are shared and debated. These projects are either newly funded, on-going or recently concluded research. The Proceedings of the 17th Italian Convention of FFC Investigators in Cystic Fibrosis report the results of the concluded research projects. Correspondence: Dr. F. Malvezzi (flaminia.malvezzi@fibrosicisticaricerca.it)

**DOI:** 10.1186/s12890-020-1104-3

**Published:** 2020-05-04

## DISEASE MODELS & PREDICTIVE TESTING

### O1 A novel Full Thickness Cystic Fibrosis model on a microfluidic chip to study pathogenic mechanisms and evaluate therapeutic strategies

#### Netti P^1^, di Bernardo D^2^

##### ^1^Centro per Biomateriali avanzati per la Sanità - CRIB, Istituto Italiano di Tecnologia, Napoli, Italy ^2^Centro di Ricerca Interdipartimentale sui Biomateriali, Università degli Studi di Napoli Federico II, Italy

###### **Correspondence:** Netti P (paoloantonio.netti@unina.it)

**Background and rationale**

Cystic Fibrosis (CF) is a highly heterogeneous disease. Several airway epithelial in vitro models have been developed to better understand pathogenic mechanisms underlying CF, to investigate the patient-specific prognosis and response to therapeutics [1]. Although these models are useful, they do not recapitulate the crosstalk between epithelial cells and the connective tissue, which has important consequences on the differentiation and function/dysfunction of the epithelium [2–4].

**Hypothesis and objectives**

The project aimed to build up a novel 3D CF model (called Full Thickness model) featured by the presence of the lung epithelial and connective compartments. Moreover, we designed and fabricated a microfluidic device for the culture of CF models, for monitoring tissue function and for administrating drugs.

**Essential methods**

The normal and CF connective airway tissues (CAT) were produced by using a bottom up approach starting from the assembly of pulmonary engineered micro-tissues. In order to build up the full thickness model, normal and CF epithelial cells were seeded on the top of the normal or CF CAT and differentiated at the Air Liquid Interface. The engineered tissues were characterized by morphological, functional and molecular analysis. The microfluidic chip was designed in Autocad and fabricated in Poly Dimethyl Siloxane using a Micromilling.

**Results**

The CF CAT showed significant differences compared to the normal one. Specifically, CF lung fibroblasts proliferated faster and produced more elements of the extracellular matrix, featured by a higher elastic modulus. Epithelial cells developed a differentiated epithelium on the surface of the CAT and penetrated the matrix forming glandular-like structures resembling submucosal glands. The viscosity of the mucus of the CF was higher than the normal model. At the same time, the microfluidic device was developed for the culture of CF models. The chip was equipped with electrodes and an aerosol for monitoring tissue function and administrating substances in the apical side.

**Conclusions**

The novel 3D model well recapitulated complications occurring during CF both in the connective and epithelial compartments. For this reason, we expect it could be used to investigate the role epithelial-stroma crosstalk in CF. Moreover, the fabricated microfluidic chip could be used for the culture of CF models, for administrating drugs in the apical or serosal side of the sample and to monitor their efficacy.

**References**

1. Ikpa P.T., Bijvelds M.J.C., De Jonge H. R. Cystic fibrosis: Toward personalized therapies. Int. J. Biochem. Cell Biol., vol. 52, pp. 192–200, 2014.

2. Casale C., Imparato G., Urciuolo F., Netti P.A. Endogenous human skin equivalent promotes in vitro morphogenesis of follicle-like structures. Biomaterials, vol. 101, pp. 86–95, 2016.

3. De Gregorio V. Imparato G, Urciuolo F, Tornesello ML, Annunziata C, Buonaguro FM, Netti PA1. An Engineered Cell-Instructive Stroma for the Fabrication of a Novel Full Thickness Human Cervix Equivalent In Vitro. Adv. Healthc. Mater., vol. 6, no. 11, 2017.

4. C. Mazio, C. Casale, G. Imparato, F. Urciuolo, and P. A. Netti. Recapitulating spatiotemporal tumor heterogeneity in vitro through engineered breast cancer microtissues. Acta Biomater., vol. 73, 2018.

**Acknowledgment** FFC#8/2017 funded by FFC and supported by Delegazione FFC di Napoli San Giuseppe Vesuviano, Delegazione FFC di Cosenza Sud, Delegazione FFC di Valle Scrivia Alessandria, Delegazione FFC di Foggia, Gruppo di Sostegno FFC di Genova "Mamme per la ricerca", Con Cecilia amici della ricerca, Gruppo di Sostegno FFC di Reggello Firenze

### O2 Phenotyping new genetically-diverse mouse models mirroring the complexity of the Cystic Fibrosis pathology

#### Lorè NI^1,2^, Sipione B^1^, Rizzo G^2^, Caslini G^1^, Ferreira ML^3^, Norata R^4^, Sitia G^3^, Rossi G^5^, Sanvito F^4^, Bragonzi A^1^

##### ^1^Infection and Cystic Fibrosis Unit, San Raffaele Scientific Institute, Milan,Italy; ^2^Vita-Salute San Raffaele University, Milan, Italy; ^3^Experimental Hepatology Unit, San Raffaele Scientific Institute, Milan, Italy; ^4^Pathological Anatomy Unit, IRCSS San Raffaele Scientific Institute, Italy; ^5^School of Biosciences and Veterinary Medicine, University of Camerino, Matelica, Italy

###### **Correspondence:** Lorè NI (lore.nicolaivan@hsr.it)

**Background**

In addition to the cystic fibrosis (CF) transmembrane conductance regulator (CFTR) gene defect, the severity of pulmonary disease correlates also to other genetic factors [1]. Several CF mouse models were generated by using redundant genetic backgrounds, whose do not represent the heterogeneity of the human population [2]. Thanks to a previous FFC project (FFC# 11/2015), we generated two new CF mouse models (CC06_CFTR^tm1kth^ and CC037_CFTR^tm1kth^) carrying the ΔF508 mutation in the different genetic background derived from the Collaborative Cross (CC) mouse population [3,4].

**Hypotesis and objectives**

The hypothesis guiding our proposal is that the mutation of CFTR in murine populations with genetic diversity may cause different pathological alterations not detected so far. The final objective is to characterize the disease manifestations in the two new CC_CF murine models.

**Methods**

CC_CF murine lines have been characterized for spontaneous pathology and for susceptibility to P. aeruginosa lung infection. Disease phenotypes have been dissected by monitoring survival rate, body weight, hematological analysis and multiorgans histopathology.

**Results**

Breeding of CC06_ΔF508-/+ heterozygotes does not produce homozygotes mice CC06_ ΔF508^-/-^ indicating potential premature death in utero for this line. However, CC37_ ΔF508^-/+^ produces 10% of vital homozygotes mice CC37_ ΔF508^-/-^. CC37_ ΔF508^-/-^ mice (CF) showed a significantly lower survival rate post birth with reduced body weight in comparison to the congenic WT mice. Hematological analysis showed significantly higher neutrophil in CF than WT, indicating an ongoing systemic inflammation. Histopathology indicated that CF mice exhibit both the expected CF-related gut pathology and, different from previous models, alterations in the lungs. Bronchial epithelium of CF mice was diffusely hyperplastic with multiple-layers of epithelial and increased of goblet cells. Muco-obstructive alterations in the lungs were observed in the trachea and major bronchi of CF mice. Additional pathological phenotypes have been observed in other organs including pancreas, heart, reproductive tract, spleen, thymus and bone marrow.

**Conclusions**

CC lines greatly expanded the range of disease phenotypes relative to classical inbred strains and may lead to the generation of diseasespecific mouse model that potentially reproduce the CF human disease.

**Reference**s

1. Cutting GR. Cystic fibrosis genetics: from molecular understanding to clinical application. Nat Rev Genet. 2015;16:45-56.

2. Zeiher BG, Eichwald E, Zabner J, Smith JJ, Puga AP, McCray PB Jr, Capecchi MR, Welsh MJ, Thomas KR. A mouse model for the delta F508 allele of cystic fibrosis. J Clin Invest. 1995;96:2051-64.

3. Lore NI, Iraqi FA, Bragonzi A. Host genetic diversity influences the severity of Pseudomonas aeruginosa pneumonia in the Collaborative Cross mice. BMC Genet. 2015;16:106.

4. Loré NI, Cigana C, Sipione B, Bragonzi A. The impact of host genetic background in the Pseudomonas aeruginosa respiratory infections. Mamm Genome. 2018 Aug;

**Acknowledgment** FFC#4/2017 funded by FFC and supported by Delegazione FFC di Milano

## ALTERNATIVE ANTIMICROBIAL STRATEGIES

### O3 Drug repurposing for antivirulence therapy against *Pseudomonas aeruginosa*

#### Leoni L^1^, Rampioni G^1^, Baldelli V^1^, Mellini M^1^, Fortuna A^1^, Bragonzi A^2^

##### ^1^ Microbial Biotechnology Lab. Department of Science, University Roma Tre, Italy; ^2^Immunology, Transplantation and Infectious Diseases, Infections and Cystic Fibrosis Unit, HSR Institute, Milan, Italy

###### **Correspondence:** Leoni L (leoni@uniroma3.it)

**Background and rationale**

The application of anti-virulence drugs to treat chronic lung infections caused by Pseudomonas aeruginosa in cystic fibrosis (CF) patients has been hampered so far by toxicity issues and limited knowledge about their efficacy on CF strains. The state of the art knowledge about P. aeruginosa infection in CF highlights three notions relevant for CF research: i) the drug repurposing approach can be used for the identification of anti-virulence drugs with low toxicity and high probability of a rapid transfer to the clinic; ii) any new compound active against P. aeruginosa model (non-CF) strains should be proven to be active also against a large proportion of strains isolated from CF patients, before further development for CF therapy; iii) it is worth to test anti-virulence drugs targeting the quorum sensing system of P. aeruginosa (pqs) for their application to CF therapy.

**Hypothesis and objectives**

We have discovered a new anti-pqs activity in three “old” FDA-approved drugs originally developed for the treatment of diseases different from P. aeruginosa infection. The possibility of repurposing these drugs to CF therapy was evaluated, also in combination with antibiotics.

**Essential methods**

The anti-virulence activity of each one of the three anti-pqs drugs was tested against a collection of 100 P. aeruginosa strains isolated from CF patients, having different antibiotic resistance profiles. The interaction of the anti-pqs drugs with antibiotics commonly used in CF therapy was studied in P. aeruginosa liquid and biofilm cultures.

**Results**

The anti-pqs drugs were significantly active against a large percentage of CF strains, even if to different extents, and showed no antagonistic effects toward antibiotics. In particular, multidrug resistant (MDR) strains seemed to be particularly susceptible to one of these drugs, named clofoctol.

**Conclusions**

This comparative analysis of three anti-pqs drugs, together with toxicity and pharmacokinetic considerations, support clofoctol as the most promising antivirulence drug for repurposing in CF therapy. However, additional confirmatory studies carried out with a larger number of MDR P. aeruginosa CF isolates should be carried out before proceedings with further studies in animal infection models.

**References**

1. Mellini M, Di Muzio E, D'Angelo F, Baldelli V, Ferrillo S, Visca P, Leoni L, Polticelli F, Rampioni G. In silico Selection and Experimental Validation of FDA-Approved Drugs as Anti-quorum Sensing Agents. Front Microbiol. 2019 Oct 10;10:2355. doi: 10.3389/fmicb.2019.02355. eCollection 2019.

2. D'Angelo F, Baldelli V, Halliday N, Pantalone P, Polticelli F, Fiscarelli E, Williams P, Visca P, Leoni L, Rampioni G. Identification of FDA-Approved Drugs as Antivirulence Agents Targeting the pqs Quorum-Sensing System of Pseudomonas aeruginosa. Antimicrob Agents Chemother. 2018 Oct 24;62(11). pii: e01296-18. doi: 10.1128/AAC.01296-18.

**Acknowledgment** FFC#17/2018 funded by FFC and supported by Delegazione FFC di Sassari con Gruppo di Sostegno FFC di Siniscola Nuoro

### O4 A longitudinal metagenomic analysis to uncover microbial signatures of CF lung disease: unravelling host-microbial community interactions in humans and animal models

#### Bevivino A^1^, Mengoni A^2^, Segata N^3^

##### ^1^Department for Sustainability, Italian National Agency for New Technologies, Energy and Sustainable Economic Development, ENEA, Rome, Italy; ^2^Department of Biology, University of Florence, Sesto Fiorentino, Florence, Italy; ^3^Centre for Integrative Biology, University of Trento, Trento, 38122, Italy

###### **Correspondence:** Bevivino A (annamaria.bevivino@enea.it)

**Background and rationale**

Although changes in the CF microbiota composition around the time of exacerbations have been described [3], the analysis of taxonomic assessment of CF lung microbiome and its functional potential have not been investigated yet [1]. In addition, no data are present on animal models, which are crucial to complement human data and delineate mechanisms of microbiome dynamics and also assist in the development of new therapies to treat patients with CF [2].

**Hypothesis and objectives**

The general aim of this proposal is to provide a more in-depth understanding of the lung microbiome in humans and animal models. The specific aims are: understand and describe the taxonomic and functional gene dynamics of the lung microbiome of CF patients; evaluate the combined effect of *Cftr* mutation and infection by *P. aeruginosa* on gut-lung microbiome in wild type and CF mice.

**Essential Methods**

Twenty-two subjects with CF, with a severe/moderate pulmonary disease, were followed over a 15-month period. Functional and taxonomic features of bacterial airway microbiome of CF patients were inferred from shotgun metagenomic data obtained from sputum samples. Also, male Cftr tm1UNCTgN(FABPCFTR) and their WT congenic mice were sacrificed at seven days post-infection to track changes of the gut and lung microbiome during chronic infection.

**Results**

Microbial strain-level population structure from metagenomes revealed a constant strain-level signature of a subject’s microbiome over time, suggesting the substantial longitudinal strain retention within the same microbial community. Time and exacerbation events impacted the microbiota dynamic from both a functional and a taxonomical perspective though the subject effect was highly relevant. CFTR-deficient and WT congenic mice do not cluster separately for lung following *P. aeruginosa* chronic infection, while a separation of the gut microbiota with respect to the mutation was found, suggesting that the CFTR genotype has more influence in our animal model for the gut microbiota than for lung microbiota.

**Conclusions**

The lung microbiome of CF patients showed an extraordinary resilience of the main CF pathogens with patient-specific colonization even at strain- level. Genes associated to metabolic pathways (including antibiotic-resistance genes) were less variable but highly patient-specific suggesting the need for future development of personalized therapeutic approaches based on patient- specific airways microbiome. The possibility to analyze the microbiome dynamics in CF airways will permit to discover novel biomarkers involved in the pulmonary disease dynamics and can give us a set of tools to unlock the potential of microbiome-based personalized medicine in major disease areas including CF [1]. Animal studies will also assist in the development of microbiome manipulation of lung microbiome aimed to restore “healthy” microbial communities.

**References**

1. Bevivino A, Bacci G, Drevinek P, Nelson MT, Hoffman L, Mengoni A. Deciphering the ecology of cystic fibrosis bacterial communities: Towards systems-level integration. Trends Mol. Med. 2019. 25(12): 1110 – 1122.

2. McCarron A, Donnelley M, Parsons D. Airway disease phenotypes in animal models of cystic fibrosis. Respir Res. 2018. 19:54. doi:10.1186/s12931-018-0750-y.

3. Carmody LA, Zhao J, Schloss PD, Petrosino JF, Murray S, Young VB, Li JZ, LiPuma JJ Changes in cystic fibrosis airway microbiota at pulmonary exacerbation. Ann Am Thorac Soc. 2013. 10(3):179-87. doi: 10.1513/AnnalsATS.201211-107OC.

**Acknowledgment** FFC#19/2017 funded by FFC and supported by Delegazione FFC Lago di Garda con i Gruppi di Sostegno FFC di Chivasso, dell'Isola Bergamasca, di Arezzo

## LUNG TRANSPLANTATION

### O5 Extracorporeal photopheresis as induction therapy to prevent acute rejection after lung transplantation in cystic fibrosis patients

#### Nosotti M^1^, Righi I^1^, Trabattoni D^2^, Clerici M^2^, Ibba S^2^, Fenizia C^2^, Morlacchi L^3^, Rossetti V^3^, Torretta L^4^, Mocellin C^4^, Prati L^4^ , Lazzari L^5^, Buono G^5^, Blasi F^3^

##### ^1^Thoracic Surgery and Lung Transplant Unit Fondazione IRCCS Ca’ Granda Ospedale Maggiore Policlinico, Milan, Italy; ^2^Laboratorio di Immunologia, Dipartimento di Scienze Biomediche Cliniche L. Sacco, Università degli Studi di Mlano, Italy; ^3^Pneumology and Respiratory Medicine Unit, Fondazione IRCCS Cà Granda Ospedale Maggiore Policlinico, Milano, Italy; ^4^Trasfusional and Apheresis Unit, Fondazione IRCCS Cà Granda Ospedale Maggiore Policlinico, Milano, Italy; ^5^Cell Factory Unit of Regenerative Medicine, Fondazione IRCCS Cà Granda Ospedale Maggiore Policlinico, Milano, Italy

###### **Correspondence:** Nosotti M (mario.nosotti@unimi.it)

**Background and rationale**

Acute rejection (AR) is common in the first year after lung transplantation (LTx). AR has usually been reversible with treatment, but it can trigger chronic rejection (CR) that is the leading causes of late morbidity and mortality. Extracorporeal photopheresis (ECP) has emerged as a promising treatment for chronic rejection. Patients affected by cystic fibrosis (CF) are more likely exposed to infections after LTx, and this can lead to episodes of rejections, both acute and chronic.

**Hypothesis and objectives**

This study aims to verify, in recipients affected with cystic fibrosis, whether the induction therapy with ECP can decrease the rate of AR, in order to impact positively on chronic rejection, that is the main cause of mortality in LTx (primary end points: survival, AR). Moreover, the safety and efficacy of ECP will be evaluated and also the infections rate. The expected results are the reduction of acute rejection episodes in its clinical, histopathologic and humoral manifestations.

**Essential methods**

This is a pilot study, single center, randomized controlled, single blind, two parallel arms: 12 patients are in the study group (ECP and standard therapy) and 12 patients are in the control group (standard therapy). Analyses of immune cell functions (in presence/absence of polyclonal or allo-specific stimulus), inflammatory status and vescicles were performed on blood and bronchoalveolar lavage at different time points in the first year after LTx. AR episodes and infections will be recorded clinically, hystologically and microbiologically. We recorded also every adverse event.

**Results**

The study started on 15^th^ September 2018, and since then we enrolled 12 patients. Preliminary data on the first ten patients (6 ECP and 4 CTR) are reported. There was one drop off due to psychological difficulties. One patient in the control group died for sepsis due to pneumonia on 10^th^ postop day. No AR episodes were observed and no adverse events due to ECP were recorded. Immune parameters were evaluated at different time points in the first year after LTx. In ECP compared to CTR patients: 1) regulatory T cells (Treg) as well as IL10 production by Treg were increased; 2) IL17-secreting Th17 as well as Th1 T cells were reduced; 3) CD107+/CD8+ (perforin-releasing CTL) T lymphocytes were reduced, 4) IL10-producing NK cells were increased; 5) LPS-stimulated IL-10 production was augmented whereas that of TNFα and IL-1β was reduced. The expression of EV-associated markers CD63, CD9 and CD81 was detected. Upregulation of platelet (CD62p; p<0.05 by t-test), lymphocytes (CD3, CD24) markers and integrins (CD29, CD49e) was observed in ECP-treated patient compared to the control group

**Conclusions**

This study is still into enrolling phase; the current interim analysis did not show any adverse effect in the study group. The immunological tests demonstrated a profound immunoregulation in the treated patients. We estimate to finish the enrolment before September 2020.

**References**

1. Aubin F, Mousson C. Ultraviolet light-induced regulatory (suppressor) T cells: an approach for promoting induction of operational allograft tolerance? Transplantation. 2004 Jan 15;77(1 Suppl):S29-31. Review. PubMed 14726767

2. Schwartz J Winters JL, Padmanabhan A, et al. Guidelines on the use of therapeutic apheresis in clinical practice-evidence-based approach from the Writing Committee of the American Society for Apheresis: the sixth special issue. J Clin Apher. 2013 Jul;28(3):145-284. doi: 10.1002/jca.21276. PubMed 23868759

3. P. Jaksch, G. Murakoezy, C. Lambers, et al. The journal of heart and lung transplantation – ISHLT 2014 Congress, Poster Session: Extracorporeal photoimmune therapy (ECP) with UVADEX in conjunction with standard therapy compared to standard therapy alone for the prevention of rejection in lung transplantation patients.

**Acknowledgment** FFC#24/2017 funded by FFC and supported by Delegazione FFC di Como Dongo, Delegazione FFC di Belluno con Rocciatori Fonzaso, Delegazione FFC di Pesaro con il Gruppo di Sostegno FFC di Fidenza

### O6 Identification of early molecular biomarkers of acute and chronic rejection in cystic fibrosis patients with lung transplant through the application of omics technologies

#### Rea F^1^, Paolo Schena F^2^

##### ^1^Dip. Scienze Cardiologiche, Toraciche e Vascolari, Div. Chirurgia toracica, AOU Padova, Italy ^2^Schena Foundation – Omics Research, Italy

###### **Correspondence:** Rea F (federico.rea@unipd.it)

**Background and rationale**

About 16% of patients undergoing lung transplant (LT) account for of end-stage cystic fibrosis (CF) patients. The first cause of LT failure is the development of acute rejection (AR) and then chronic lung allograft dysfunction (CLAD) that affects 50% of patients at 5 years post-transplantation. CLAD includes two entities: bronchiolitis obliterans syndrome and restrictive allograft syndrome. Both phenotypes have poor prognosis with a median survival of less than 2 years for RAS and less than 4 years for BOS. Since CLAD is irreversible, it is necessary to study the molecular mechanisms leading to such a condition with the ultimate goal of identifying early biomarkers for diagnosis and targeted therapy.

**Hypothesis and Objective**

Our project is the molecular study of rejection in CF patients that undergone lung transplant by performing RNA-Seq in transbronchial biopsy (TBB) specimens.

**Essential methods**

From August 2015 a certified biobank is available at the Pathological Anatomy Unit of the Padova University Hospital. For each of the follow-up time-points, TBB have been stored according to specific guidelines in order to perform omics analyses. Illumina NextSeq 500 has been used for sequencing RNA obtained from formalin-fixed paraffin-embedded (FFPE) TBB specimens. The quality of RNA has been checked before RNA-seq that has been sequenced in pair-end mode. Prior to further analysis, a quality check of the sequencing data has been performed.

**Preliminary Results**

In this retrospective study we have enrolled 18 patients that undergone lung transplant for end-stage CF and 10 cadaveric lung donors. TBBs have been evaluated for the presence of AR and CLAD according to the guidelines of the ISHLT. Transcriptomics has been performed in 40 TBB samples (11 CLAD, 6 AR, 13 samples with no rejection and 10 donor lungs obtained before transplantation). Statistical analysis, performed by the Bioconductor tool, generated a gene list of 7675 probes corrected with p<0.05 for the AR and 4605 for the CLAD. Ingenuity Pathway Analysis (IPA) software that assesses biological relationships among genes and entities with a FC>1.5 evidenced 5 different pathways and 3 genes (CACNA1I, CERS3, FLG) that were common in AR and CLAD. Real-time PCR to validate differential gene expression data and immunohistochemistry on lung tissue specimens are in progress.

**Conclusions**

Integrating histological data and transcriptomics we may provide more information on the molecular mechanisms of AR and CLAD in end-stage CF patients with lung transplant.

**Acknowledgment** FFC#28/2018 funded by FFC and supported by Delegazione FFC di Boschi Sant'Anna Minerbe

## POSSIBLE TARGETS AND MECHANISMS OF CFTR MODULATORS

### O7 Proteomic profiling of F508del-CFTR cells to identify new pharmacological targets for CF

#### Braccia C^1,4^, Tomati V^2^, Liessi N^3^, Pedemonte N^2^ and Armirotti A^3^

##### ^1^D3Pharmachemistry, Fondazione Istituto Italiano di Tecnologia, Via Morego 30, 16163 Genova, Italy; ^2^U.O.C. Genetica Medica, IRCCS Istituto Giannina Gaslini, Via Gerolamo Gaslini 5, 16147 Genova, Italy; ^3^Analytical Chemistry Lab, Fondazione Istituto Italiano di Tecnologia, Via Morego 30, 16163 Genova, Italy; ^4^Dipartimento di Chimica, Università degli Studi di Genova, Via Dodecaneso 31, 16146 Genova, Italy

###### **Correspondence:** Armirotti A (andrea.armirotti@iit.it)

**Background and Rationale**

An approach to identify new targets for CF is the screening of siRNA libraries designed to silence molecules with a role in F508del-CFTR processing and degradation, in order to pinpoint those that, upon inhibition, lead to CFTR rescue at the plasma membrane [1]. This screening, performed at Gaslini Hospital by Pedemonte’s group, already allowed the identification of some new candidate targets [1,2]. Still, some of the identified molecules (primary targets) are “big-players” in the cell machinery and their inhibition might potentially arise safety concerns.

**Hypothesis and objectives**

In the frame of first year project, we quantified more than 4000 proteins in the bronchial epithelium of CF patients, and we found that 154 of them are significantly dysregulated by the disease [3]. We then applied the same technique to CFBE41o- cells, following the abolition of the four primary targets (FAU, RNF5, LRRC59 and PHF12).

**Essential methods**

We performed the proteomic analysis by using SWATH-MS [4] and a dedicated protocol optimized for the proteomic investigation of the human bronchial epithelium. We used publicly available software tools to annotate the functions of the dysregulated proteins and to investigate protein networks.

**Results**

Our results showed that changes in protein expression are clearly detectable after the silencing of the secondary targets, thus supporting the feasibility of our project. Indeed, we identified seven and eleven proteins that are respectively up- and down-regulated following the abolition of the primary targets. We thus consider these proteins as potential secondary targets. We also identified four biological pathways that are significantly downregulated by the same CFTR rescuing maneuvers. Keeping safety concerns in mind, we then selected a number of secondary targets and some of the key proteins for the downregulated pathways and we silenced them in CFBE41o- models. Very promisingly, some of these proteins triggered a significant rescue of CFTR.

**Conclusions**

With the use of proteomics and bioinformatics, we managed to identify a set of proteins (secondary targets) that, when silenced in CFBE410-, trigger a significant rescue of CFTR. The challenge is now to translate these results to primary cell cultures from CF patients, by modulating these targets by mean of commercially available pharmacological modulators.

**References**

1. Tomati V, Pesce E, Caci E, Sondo E, Scudieri P, Marini M, Amato F, Castaldo G, Ravazzolo R, Galietta LJV, Pedemonte N. High-throughput screening identifies FAU protein as a regulator of mutant cystic fibrosis transmembrane conductance regulator channel. J Biol Chem. 2018 Jan 26;293(4):1203-1217. doi: 10.1074/jbc.M117.816595.

2. Tomati V, Sondo E, Armirotti A, Caci E, Pesce E, Marini M, Gianotti A, Jeon YJ, Cilli M, Pistorio A, Mastracci L, Ravazzolo R, Scholte B, Ronai Z, Galietta LJ, Pedemonte N. Genetic Inhibition Of The Ubiquitin Ligase Rnf5 Attenuates Phenotypes Associated To F508del Cystic Fibrosis Mutation. Sci Rep. 2015 Jul 17;5:12138. doi: 10.1038/srep12138.

3. Braccia C, Tomati V, Caci E, Pedemonte N, Armirotti A. SWATH label-free proteomics for cystic fibrosis research. J Cyst Fibros. 2019 Jul;18(4):501-506. doi: 10.1016/j.jcf.2018.10.004. Epub 2018 Oct 19.

4. Kang Y, Burton L, Lau A, Tate S. SWATH-ID: An instrument method which combines identification and quantification in a single analysis. Proteomics. 2017 May;17(10):e1500522. doi: 10.1002/pmic.201500522.

5. Rosenberger G, Koh CC, Guo T, Röst HL, Kouvonen P, Collins BC, Heusel M, Liu Y, Caron E, Vichalkovski A, Faini M, Schubert OT, Faridi P, Ebhardt HA, Matondo M, Lam H, Bader SL7, Campbell DS, Deutsch EW, Moritz RL, Tate S, Aebersold R. A repository of assays to quantify 10,000 human proteins by SWATH-MS. Sci Data. 2014 Sep 16;1:140031. doi: 10.1038/sdata.2014.31. eCollection 2014.

**Acknowledgment** FFC#1/2018 funded by FFC and supported by Delegazione FFC di Genova e Gruppo di Sostegno FFC di Savona Spotorno

### O8 Identification of deubiquitinases and ubiquitin ligases that affect mutant CFTR rescue

#### Musante I, Renda M, Venturini A, Galietta LJV

##### Telethon Institute of Genetics and Medicine, TIGEM, Pozzuoli, Italy

###### **Correspondence:** Galietta LJV (l.galietta@tigem.it)

**Background and Rationale**

F508del, the most frequent mutation in cystic fibrosis (CF), impairs the folding and stability of the CFTR chloride channel [1]. Despite the treatment with pharmacological correctors a significant fraction of mutant CFTR (F508del-CFTR) is eliminated by cell quality control mechanisms based on ubiquitination and proteasome/lysosome-dependent degradation [2].

**Hypothesis and Objectives**

Our proposal aims at the identification of deubiquitinases (DUBs) and ubiquitin ligases (UBLs) that control mutant CFTR degradation. Identification of the main mechanisms responsible for mutant CFTR degradation, and development of pharmacological strategies to contrast them, may help to achieve better levels of CFTR correction.

**Essential Methods**

We used pharmacological modulators and gene silencing to identify the DUBs that are involved in F508del-CFTR processing.

**Results**

Using gene silencing by siRNA transfection, we have identified a panel of DUBs that influence F508del-CFTR rescue by corrector VX-809. In particular, knockdown of USP13 results in decreased F508del-CFTR function [3]. Therefore, these DUBs may have a protective role on F508del-CFTR by contrasting the process of ubiquitination that occurs even in the presence of the corrector. Intriguingly, we also found DUBs whose silencing amplifies mutant CFTR rescue. In this case, we need to postulate a more indirect mechanism in which the DUB activity affects the expression/function of a regulator of F508del-CFTR processing.

**Conclusions**

A better knowledge of the mechanisms that limit mutant CFTR rescue can lead to improved therapeutic strategies.

**References**

1. Lukacs GL, Verkman AS. CFTR: folding, misfolding and correcting the ΔF508 conformational defect. 2012. Trends Mol Med 18: 81-91

2. He L, Kota P, Aleksandrov AA, Cui L, Jensen T, Dokholyan NV, Riordan JR. Correctors of deltaF508 CFTR restore global conformational maturation without thermally stabilizing the mutant protein. 2013. FASEB J 27: 536-545

3. Pesce E, Sondo E, Ferrera L, Tomati V, Caci E, Scudieri P, Musante I, Renda M, Baatallah N, Servel N, Hinzpeter A, di Bernardo D, Pedemonte N, Galietta LJV. The autophagy inhibitor spautin-1 antagonizes rescue of mutant CFTR through an autophagy-independent and USP13-mediated mechanism. 2018. Front Pharmacol 9:1464

**Acknowledgment**. FFC#2/2017 funded by FFC and supported by Delegazione FFC di Bologna e Delegazione FFC di Ferrara con il Gruppo di sostegno FFC di Comacchio

### O9 Dissecting the mechanism of action of the TG2 inhibitor cysteamine on Cystic Fibrosis

#### Piacentini M^1^, Maiuri L^2^, Delogu G^3^, Rossin F^3^

##### ^1^Università Roma Tor Vergata, Dip. Biologia, Italy; ^2^Istituto Europeo Ricerca Fibrosi Cistica - IERFC c/o Istituto San Raffaele, Milano,Italy; ^3^Università Cattolica del Sacro Cuore, Fondazione Policlinico Gemelli, Istituto di Microbiologia, Roma, Italy

###### **Correspondence:** Piacentini M (mauro.piacentini@uniroma2.it)

**Background/Rationale**

Transglutaminase 2 (TG2), the most ubiquitous member of the TG family, plays a crucial role in Cystic Fibrosis (CF) pathogenesis. TG2 is a multifunctional enzyme involved in a variety of cellular processes by playing a key regulatory role in intracellular proteostasis under stressful conditions. TG2 catalyses post-translational modifications of proteins through both Ca^2+^ dependent and independent reactions. In fact, in addition to its crosslinking activity, TG2 may also act as protein disulphide isomerase [1]. It has been demonstrated that TG2 is constitutively up regulated in CF airways and drives chronic inflammation. The enzyme deregulation in CF disrupts the TG2 mediated capability of fighting stress, making TG2 a harmful, instead of beneficial, player of the disease pathogenesis [2]. Several TG2 inhibitors can ameliorate the disease phenotype such as cysteamine, a small molecule with pleiotropic functions, among which the capability of controlling TG2 overactivation in CF improving the trafficking and the function of F508del CFTR [3].

**Hypothesis and Objectives**

The aim of this project is to elucidate the molecular mechanisms by which cysteamine modulates CFTR trafficking and consequently the susceptibility to opportunistic airways infections. We will assess the activity of cysteamine against bacterial infection and the effect on the activation of the innate immune response analysing the STING pathway in the CF models. Moreover, we will perform a transcriptome sequencing in human and mouse CF models using cysteamine to inhibit TG2 with the aim to obtain a platform of new possible CF targets.

**Essential methods**

We will use CF mouse models infected in vivo and ex vivo with Mabs and P. aeruginosa as well as peripheral blood mononuclear cell from CF patients.

**Results**

Our findings indicate that cysteamine can improve the clearance of other pathogenic mycobacteria such as Mycobacterium abscessus. Moreover, we found that TG2 is able to control the innate immune response by regulating type 1 interferon production, thus possibly explaining the negative role of the enzyme in the infection process.

**Conclusions**

The results of this project will define the molecular basis that supports the use of cysteamine not only as a CFTR corrector but also as a promising therapy against bacterial opportunistic infections [4, 5]. To understand the molecular pathway involved in bacterial infection could provide new possible targets and the possibility to define novel strategies aimed to improve the health care of CF patients.

**Reference**

1. Rossin F, Villella VR, D'Eletto M, Farrace MG, Esposito S, Ferrari E, Monzani R, Occhigrossi L, Pagliarini V, Sette C, Cozza G, Barlev NA, Falasca L, Fimia GM, Kroemer G, Raia V, Maiuri L, Piacentini M. 2018. TG2 regulates the heat-shock response by the post-translational modification of HSF1. EMBO Rep. 19.

2. Luciani, A., Villella, V. R., Esposito, S., Brunetti-Pierri, N., Medina, D., Settembre, C., Gavina, M., Pulze, L., Giardino, I., Pettoello-Mantovani, M., D'Apolito, M., Guido, S., Masliah, E., Spencer, B., Quaratino, S., Raia, V., Ballabio, A. and Maiuri, L. (2010). Defective CFTR induces aggresome formation and lung inflammation in cystic fibrosis through ROS-mediated autophagy inhibition. Nat. Cell Biol. 12, 863-75.

3. Tosco, A., De Gregorio, F., Esposito, S., De Stefano, D., Sana, I., Ferrari, E., Sepe, A., Salvadori, L., Buonpensiero, P., Di Pasqua, A., Grassia, R., Leone, C. A., Guido, S., De Rosa, G., Lusa, S., Bona, G., Stoll, G., Maiuri, M. C., Mehta, A., Kroemer, G., Maiuri, L. and Raia, V. (2016). A novel treatment of cystic fibrosis acting on-target: cysteamine plus epigallocatechin gallate for the autophagy-dependent rescue of class II-mutated CFTR. Cell Death Differ. 23, 1380-93.

4. Palucci I, Matic I, Falasca L, Minerva M, Maulucci G, De Spirito M, Petruccioli E, Goletti D, Rossin F, Piacentini M, Delogu G. 2018. Transglutaminase type 2 plays a key role in the pathogenesis of Mycobacterium tuberculosis infection. J Intern Med. 283, 303-313.

5. Ferrari E, Monzani R, Villella VR, Esposito S, Saluzzo F, Rossin F, D'Eletto M, Tosco A, De Gregorio F, Izzo V, Maiuri MC, Kroemer G, Raia V, Maiuri L. 2017. Cysteamine re-establishes the clearance of Pseudomonas aeruginosa by macrophages bearing the cystic fibrosis-relevant F508del-CFTR mutation. Cell Death Dis. 8, e2544.

**Acknowledgment** FFC#10/2018 funded by FFC and supported by Delegazione FFC di Napoli San Giuseppe Vesuviano e Associazione “Gli amici della Ritty" Casnigo

## POSSIBLE NEW MODULATORS OF MUTANT CFTR

### O10 RNF5 inhibitors as potential drugs for Cystic Fibrosis basic defect

#### Pedemonte N^1^, Cavalli A^2^

##### ^1^U.O.C Genetica Medica, Istituto Giannina Gaslini, Genova; ^2^Dipartimento di Farmacia e Biotecnologie, Università di Bologna (FFC#9/2017)

###### **Correspondence:** Pedemonte N (nicoletta.pedemonte@unige.it)

**Background and rationale**

The F508del mutation causes the arrest of the maturation of CFTR protein. Correctors are able to rescue F508del-CFTR, either by act directly on CFTR or by modulating other proteins thus affecting CFTR maturation. Our studies highlighted the E3 ubiquitin ligase RNF5 [1] as a target whose inhibition leads to mutant CFTR rescue both in vitro and in vivo [2]. Thus, we used a computational approach, based on ligand docking and virtual screening, to discover inh-02, a drug-like small molecule that inhibits RNF5 [3].

**Hypothesis and objectives**

Treatment with inh-02 causes significant F508del-CFTR rescue in immortalized and primary bronchial epithelial cells from F508del homozygous CF patients. Our aims now are: 1. the optimization of RNF5 inhibitors; 2. the evaluation of possible individual variability in the efficacy of RNF5 inhibitors; 3. the evaluation of possible toxicity of RNF5 inhibitors (due to their mechanism of action).

**Essential methods**

Improved RNF5 inhibitors will be developed by screening commercially available analogs and by synthesizing novel analogs. The knowledge of the structure-activity relationship will help us to improve efficacy and potency of RNF5 inhibitors. The efficacy of RNF5 inhibitors on mutant CFTR will be assessed by electrophysiological techniques on bronchial epithelia derived from patients bearing F508del or other mutations with trafficking defect.

**Results**

We have tested a set of inh-2 analogs and identified moieties that are mandatory for the activity of the compounds. We have verified the ability of inh-2 to rescue F508del-CFTR on well-differentiated primary cultures of human bronchial epithelial cells from various F508del homozygous subjects. We have observed lack of side effects after long-term treatment of bronchial cells with inh-2. We have demonstrated that inh-2 is additive with both C2 and C3 types of correctors [4].

**Conclusions**

Our results clearly demonstrate that RNF5 inhibition can rescue F508del-CFTR trafficking defect and that this mechanism is not only amenable in cell lines or in a murine CF model, but also in human primary bronchial epithelia, that are the main target tissue of CF treatment. These findings thus validate RNF5 as a drug target for CF, and provide evidences to support its druggability.

**References**

1. Younger JM1, Chen L, Ren HY, Rosser MF, Turnbull EL, Fan CY, Patterson C, Cyr DM. Sequential quality-control checkpoints triage misfolded cystic fibrosis transmembrane conductance regulator. Cell. 2006 Aug 11;126(3):571-82.

2. Tomati V, Sondo E, Armirotti A, Caci E, Pesce E, Marini M, Gianotti A, Jeon YJ, Cilli M, Pistorio A, Mastracci L, Ravazzolo R, Scholte B, Ronai Z, Galietta LJ, Pedemonte N. Genetic Inhibition Of The Ubiquitin Ligase Rnf5 Attenuates Phenotypes Associated To F508del Cystic Fibrosis Mutation. Sci Rep. 2015 Jul 17;5:12138. doi: 10.1038/srep12138.

3. Sondo E, Falchi F, Caci E, Ferrera L, Giacomini E, Pesce E, Tomati V, Mandrup Bertozzi S, Goldoni L, Armirotti A, Ravazzolo R, Cavalli A, Pedemonte N. Pharmacological Inhibition of the Ubiquitin Ligase RNF5 Rescues F508del-CFTR in Cystic Fibrosis Airway Epithelia. Cell Chem Biol. 2018 Jul 19;25(7):891-905.e8. doi: 10.1016/j.chembiol.2018.04.010. Epub 2018 May 10.

4. Veit G, Xu H, Dreano E, Avramescu RG, Bagdany M, Beitel LK, Roldan A, Hancock MA, Lay C, Li W, Morin K, Gao S, Mak PA, Ainscow E, Orth AP, McNamara P, Edelman A, Frenkiel S, Matouk E, Sermet-Gaudelus I, Barnes WG, Lukacs GL. Structure-guided combination therapy to potently improve the function of mutant CFTRs. Nat Med. 2018 Nov;24(11):1732-1742. doi: 10.1038/s41591-018-0200-x. Epub 2018 Oct 8.

**Acknowledgment**. FFC#9/2017 funded by FFC and supported by Delegazione FFC di Genova e Gruppo di Sostegno FFC di Savona Spotorno, "Un fiore per Valeria" Assemini – Cagliari, Gruppo di Sostegno FFC di Vigevano, Delegazione FFC della Valdadige, Delegazione FFC di Lodi

### O11 Rescuing defective CFTR applying a drug repositioning strategy based on computational studies, surface plasmon resonance and cell-based assays

#### D’Ursi P^1^, Orro A^1^, Urbinati C^2^, Uggeri M^1^, Paiardi G^2^, Millo E^3,4^, Milanesi L^1^, Pedemonte N^5^ , Fossa P^6^ and Rusnati M^1^

##### ^1^ITB-CNR Segrate Milano, Italy; ^2^DMMT University of Brescia, italy; ^3^Med Sperim. University of Genoa, Italy; ^4^CEBR, University of Genova, Italy; ^5^UOC Genetica Medica, IRCCS Giannina Gaslini Inst. Genova, Italy; ^6^Dipartimento di Farmacia, University of Genova, Italy

###### **Correspondence:** Rusnati M (marco.rusnati@unibs.it)

**Background**

Cystic Fibrosis (CF) is caused by mutations (mainly F508del) of the cystic fibrosis transmembrane conductance regulator (CFTR). Current CF therapies are aimed at symptoms alleviation, calling for new drugs to rescue CFTR function.

**Hypothesis and objectives**

Drug repositioning is aimed at finding new applications for already marketed drugs, reducing cost and duration and the likelihood of unforeseen adverse events. In this project we have integrated drug repositioning with computational studies, surface plasmon resonance (SPR) [1] and well-tried cellular models [2] to identify new CF drugs and to comprehend their mechanism of action.

**Methods and results**

We have prepared a new structural homology model of intact human F508del-CFTR embedded in a phospholipid bilayer and a SPR biosensor containing the same protein in a cell membrane-mimicking lipid film.

These tools, along with appropriate cell-based assays, have been firstly used to analyze a mixed library of well-known and new compounds that allowed the validation of the system and the identification of a promising molecule endowed with a F508del-binding and rescuing capacity that is higher than those of drugs already in use.

With the computational model we have then performed a virtual drug repositioning on a library of 846 drugs, identifying 10 drugs that were reduced to 4 on the basis of toxicity profile and patient compliance. These drugs will be now subjected to experimental analysis by cell-based and SPR assays for their effective capacity to bind F508del-CFTR and rescue its activity. Also, we will proceed to the virtual repositioning of a library of natural compounds.

**Spin-off for research & clinical purposes**

The novel computational models and biosensors will widen the study of CF drugs and made available to other research groups in the field of CF.

**References**

**1.** Rusnati M, Sala D, Orro A, Bugatti A, Trombetti G, Cichero E, Urbinati C, Di Somma M, Millo E, Galietta LJV, Milanesi L, Fossa P, D'Ursi P. Speeding Up the Identification of Cystic Fibrosis Transmembrane Conductance Regulator-Targeted Drugs: An Approach Based on Bioinformatics Strategies and Surface Plasmon Resonance. Molecules. 2018 Jan 8;23(1). pii: E120. doi: 10.3390/molecules23010120.

2. Tomati V, Pesce E, Caci E, Sondo E, Scudieri P, Marini M, Amato F, Castaldo G, Ravazzolo R, Galietta LJV, Pedemonte N. High-throughput screening identifies FAU protein as a regulator of mutant cystic fibrosis transmembrane conductance regulator channel. J Biol Chem. 2018 Jan 26;293(4):1203-1217. doi: 10.1074/jbc.M117.816595. Epub 2017 Nov 20.

**Acknowledgment** FFC#11/2018 funded by FFC and supported by Delegazione FFC di Torino

## GENE AND RNA EDITING

### O12 Investigating CRISPR-CAS13b as a tool for the RNA editing of CFTR mRNA with premature stop codon

#### Di Leonardo A, Melfi R, Cancemi P, Chiavetta R

##### Department of Biological, Chemical and Pharmaceutical Sciences and Technologies, University of Palermo, Italy*.*

###### **Correspondence:** Di Leonardo A (aldo.dileonardo@unipa.it)

**Background and Rationale**

Some CF patients are compound heterozygous or homozygous for nonsense mutations in the CFTR gene. Mutant CFTR gene coding for transcripts with premature termination codons (PTCs) is responsible for truncated CFTR protein and for a severe form of the disease. In a precision medicine framework the *“REPAIRv2” (RNA Editing for Programmable A to I Replacement v2*) tool, developed in the laboratory of Dr. Feng Zhang (USA), seems a good alternative to restore the full-length CFTR protein by editing its mRNA containing PTCs. This new approach is based on the possibility of targeting a deaminase enzyme (huADAR2) to a specific Adenosine, to be edited to Inosine (G analogue), on the mutant RNA by a specific guide RNA (gRNA), complementary to the target regions, and a Cas protein.

**Hypothesis and objectives**

We applied the new CRISPR/dCas13b based molecular tool of RNA editing (REPAIRv2) to correct the premature stop codon UGA, changing to UGG, in the H2bGFP*opal* and CFTR^W1282X^ mRNAs with the purpose of recovering the full-length proteins.

**Essential Methods**

We designed and cloned the gRNAs needed to target the REPAIRv2 system to the Adenine to be modified. By site-directed mutagenesis we introduced a premature stop codon, W1282X, in the CFTR cDNA. Human HeLa cells expressing the H2BGFPopal mRNA, FRT cells expressing CFTR^W1282X^ and IB3.1 airway epithelial human cells (CFTRΔ508/W12382X) were co-transfected with the plasmids coding for the recombinant protein dCAS13b/ADAR2_DD_, and for the gRNAs. Fluorescence microscopy was used to analyse the editing results.

**Results**

Direct fluorescence microscopy and immunofluorescence analyses detecting the corrected proteins (H2BGFP and CFTR, respectively) suggest that the REPAIRv2 system was able, in different cell lines, to edit the H2BGFP*opal* and the CFTRW^1282X^ mRNA. However, the rate of editing does not seem high. Indeed, when RNA was purified from transfected cell, retro-transcribed and amplified base correction was not detectable by standard DNA sequencing and western blot.

**Conclusions**

Collectively, our results indicate that the *REPAIRv2* tool is able to edit the UGA premature stop codon present in the HeLa-H2BGFP*opal* cells and in engineered FRT^W1282X^ cells harbouring the UGA PTC in the CFTR mRNA. Furthermore, the *REPAIRv2* tool worked in the IB3.1 cells suggesting its ability to edit endogenous UGA premature stop codon. Anyway, enhance the delivery of the plasmids as well increase/stabilize the target mRNA to be edited, seem necessary to improve the efficiency of *REPAIRv2.*

**References**

1. Cox DBT, Gootenberg JS, Abudayyeh OO, Franklin B, Kellner MJ, Joung J, Zhang F.- RNA editing with CRISPR-Cas13. Science. 2017 Nov 24; 358 (6366):1019-1027)

2. Lentini L, Melfi R, Di Leonardo A, Spinello A, Barone G, Pace A, Palumbo Piccionello A, Pibiri I. Toward a rationale for the PTC124 (Ataluren) promoted readthrough of premature stop codons: a computational approach and GFP-reporter cell-based assay. Mol Pharm. 2014 Mar 3;11(3):653-64.

**Acknowledgment** FFC#5/2018 funded by FFC and supported by Delegazione FFC di Palermo

### O13 SpliceFix: fixing splicing defects in the CFTR gene through CRISPR/Cas9 technology

#### Giulia Maule^1^, Antonio Casini^1^, Claudia Montagna^1^, Daniele Arosio^2^, Zeger Debyser^3^, Marianne Carlon^3^, Gianluca Petris^1^, Anna Cereseto^1^

##### ^1^Centre for Integrative Biology (CIBIO), University of Trento, Via Sommarive 9, 38123 Trento, Italy; ^2^Institute of Biophysics, National Research Council and Bruno Kessler Foundation, 38122 Trento, Italy; ^3^Laboratory for Molecular Virology and Gene Therapy, Department of Pharmaceutical and Pharmacological Sciences, KU Leuven, Belgium.

###### **Correspondence:** Anna Cereseto (anna.cereseto@unitn.it)

**Background/Rationale**

A significant number of mutations (~13%) alter the correct splicing of the *CFTR* gene, causing the production of aberrant mRNA transcripts and non-functional protein channels. The 3242-26A>G and 3849+10kbC>T are point mutations that generate altered splicing [1–4]. The resulting mRNAs contain frameshifts in *CFTR*, producing a premature termination codons and consequent expression of a truncated non-functional CFTR protein. With this project we investigated an efficient genome editing approach to permanently correct this splicing defect [5].

**Hypothesis and Objectives**

The development of precise and efficient targeted nucleases has highly accelerated the progress of gene correction for genetic diseases, including Cystic Fibrosis (CF) [6]. In contrast to classical gene addition strategies, correction of the mutated *CFTR* by genome editing holds the promise to restore physiological levels of CFTR expression and function. We developed a genome editing strategy to repair 3272-26A>G and 3849+10kbC>T mutations through the exploitation of RNA guided nucleases (SpCas9 or AsCas12a) [7–9]. CFTR splicing models (minigenes) have been studied to design and develop a safe CRISPR/Cas dedicated approach aimed at restoring the correct *CFTR* gene expression. We then adapted the technology to viral vectors and applied to model organoids derived from patient’s primary cells.

**Essential methods**

Minigene constructs and cellular models were used to optimize the genome editing approach. We evaluated two approaches to edit the mutated by using two gRNAs or a single gRNA in combination with SpCas9 or AsCas12a. To test the efficacy of the genome editing method we used a novel Chloride sensor to measure CFTR activity. The correction of the splicing defect was genetically and functionally evaluated in organoids derived from patient compound heterozygous for the 3272-26A>G and3849+10kbC>T splicing mutation.

**Results**

We generated a minigene-constructs to efficiently model the 3272-26A>G *CFTR* and 3849+10kbC>T splicing defects. The analyses performed with the minigene models, either transiently or stably transfected in HEK293 cells and Caco-2 cells, in primary airway cells and in patients derived organoids revealed that the AsCas12a in combination with a selected guide RNA is ahighly efficient and precise technique to repair the splicing defects [5].

**Conclusions**

Our results demonstrate that AsCas12a in combination with a single sgRNA efficiently rescue endogenous CFTR function in patient’s intestinal organoids, which are recognised as a highly valuable preclinical model to predict *ex vivo* any success of a therapeutic treatment in human patients [10,11]. Our results provide an important milestone towards the development of a successful gene therapy clinical approach for the treatment of splicing defects in Cystic Fibrosis.

**References**

1. Beck S, Penque D, Garcia S, Gomes A, Farinha C, Mata L, Gulbenkian S, Gil-Ferreira K, Duarte A, Pacheco P, Barreto C, Lopes B, Cavaco J, Lavinha J, Amaral MD. Cystic fibrosis patients with the 3272-26A-->G mutation have mild disease, leaky alternative mRNA splicing, and CFTR protein at the cell membrane. Hum Mutat. 1999;14(2):133-44.

2. Amaral MD, Pacheco P, Beck S, Farinha CM, Penque D, Nogueira P, Barreto C, Lopes B, Casals T, Dapena J, Gartner S, et al. Cystic fibrosis patients with the 3272-26A>G splicing mutation have milder disease than F508del homozygotes: a large European study. J Med Genet. 2001 Nov;38(11):777-83.

3. Highsmith WE, Burch LH, Zhou Z, Olsen JC, Boat TE, Spock A, Gorvoy JD, Quittel L, Friedman KJ, Silverman LM, et al. A novel mutation in the cystic fibrosis gene in patients with pulmonary disease but normal sweat chloride concentrations. N Engl J Med. 1994 Oct 13;331(15):974-80.

4. Duguépéroux I, De Braekeleer M. The CFTR 3849+10kbC->T and 2789+5G->A alleles are associated with a mild CF phenotype. Eur Respir J. 2005 Mar;25(3):468-73.

5. Maule G, Casini A, Montagna C, Ramalho AS, De Boeck K, Debyser Z, Carlon MS, Petris G, Cereseto A. Allele specific repair of splicing mutations in cystic fibrosis through AsCas12a genome editing. Nat Commun. 2019 Aug 7;10(1):3556. doi: 10.1038/s41467-019-11454-9.

6. Razzouk S. CRISPR-Cas9: A cornerstone for the evolution of precision medicine. Ann Hum Genet. 2018 Nov;82(6):331-357. doi: 10.1111/ahg.12271. Epub 2018 Jul 16.

7. Knott GJ, Doudna JA. CRISPR-Cas guides the future of genetic engineering. Science. 2018 Aug 31;361(6405):866-869. doi: 10.1126/science.aat5011.

8. Kleinstiver BP, Tsai SQ, Prew MS, Nguyen NT, Welch MM, Lopez JM, McCaw ZR, Aryee MJ, Joung JK. Genome-wide specificities of CRISPR-Cas Cpf1 nucleases in human cells. Nat Biotechnol. 2016 Aug;34(8):869-74. doi: 10.1038/nbt.3620. Epub 2016 Jun 27.

9. Kim D, Kim J, Hur JK, Been KW, Yoon SH, Kim JS. Genome-wide analysis reveals specificities of Cpf1 endonucleases in human cells. Nat Biotechnol. 2016 Aug;34(8):863-8. doi: 10.1038/nbt.3609. Epub 2016 Jun 6

10. Dekkers JF, Wiegerinck CL, de Jonge HR, Bronsveld I, Janssens HM, de Winter-de Groot KM, Brandsma AM, de Jong NW, Bijvelds MJ, Scholte BJ, Nieuwenhuis EE, van den Brink S, Clevers H, van der Ent CK, Middendorp S, Beekman JM. A functional CFTR assay using primary cystic fibrosis intestinal organoids. Nat Med. 2013 Jul;19(7):939-45. doi: 10.1038/nm.3201. Epub 2013 Jun 2.

11. Dekkers JF, Berkers G, Kruisselbrink E, Vonk A, de Jonge HR, Janssens HM, Bronsveld I, van de Graaf EA, Nieuwenhuis EE, Houwen RH, Vleggaar FP, Escher JC, de Rijke YB, Majoor CJ, Heijerman HG, de Winter-de Groot KM, Clevers H, van der Ent CK, Beekman JM. Characterizing responses to CFTR-modulating drugs using rectal organoids derived from subjects with cystic fibrosis. Sci Transl Med. 2016 Jun 22;8(344):344ra84. doi: 10.1126/scitranslmed.aad8278.

**Acknowledgment**. FFC#1/2017 funded by FFC and supported by Associazione Trentina Fibrosi Cistica in ricordo di Maria Cainelli e Romana Petrolli, Delegazione FFC di Imola e Romagna, Delegazione FFC di Alberobello, Delegazione FFC di Lucca

## TARGENTING NON F508del-CFTR MUTATIONS

### O14 Optimization of a new lead promoting the readthrough of nonsense mutations for the CFTR rescue in human CF cells

#### Laura Lentini, Raffaella Melfi, Marco Tutone, Sara Baldassano, Aldo Di Leonardo, Andrea Pace and Ivana Pibiri

##### Department STEBICEF-University of Palermo – viale delle Scienze 90128, Parco d’Orleans-Ed.16 and ED.17. Italy

###### **Correspondence:** Laura Lentini (laura.lentini@unipa.it)

**Background/Rationale**

Cystic Fibrosis (CF) patients with *nonsense* (*ns*) mutations in the CFTR gene have a more severe form of the disease. A potential treatment is to promote translational readthrough of premature termination codons (PTCs) by Translational Read- Through-Inducing Drugs (TRIDs) [1].

**Hypothesis and objectives**

In our previous study, by computational and biological screening we identified a new small molecule showing high readthrough activity [2-4]. Our intent was to optimize the lead molecule by *QSAR* study and to synthetize a small library of compounds as suggested by the computational study t*o* evaluate the CFTR expression and functionality after treatments in CF model systems. Moreover we aimed to evaluate the activity of new molecules, in cells expressing a *nonsense-*CFTR-mRNA (*ns* CFTR).

Further issues considered are the toxicity of evaluated molecules in animal model (zebrafish) and mechanism of action of the designed molecules as TRIDs.

**Essential Methods**

QSAR carried out on the basis of our previous results (FFC#1/2014), was performed to achieve the lead optimization and a small library of analogs has been synthesized in order to be tested. The project was aimed to assay the CFTR functionality after treatment with NV2445 molecule and its analogs in cells harboring the most frequent *nonsense (ns)* mutations of the CFTR gene. FRT cells engineered with a vector expressing *ns*CFTR were grown in the a system to reproduce in vitro the epithelial organization. CFTR expression was evaluated by Real time RT PCR and Western blot analysis. After treatments with NV molecules , the channel functionality was measured by the EYFP quenching assay and Ussing chamber. Finally, the toxicity of two selected molecules was tested in vivo on zebrafish model.

**Results**

After virtual screening, we synthesized a small library of analogues of the NV2445 molecule and tested them by FLuc assay and in different biological models. These new compounds showed high read-through capacity and CFTR rescue.

Moreover we performed in vitro and in vivo toxicity test (Zebrafish) to assess the safety profile of our molecules.

**Conclusions**

Identification of molecules displaying readthrough activity. Selection of a lead compound as therapeutic approach to the second cause of FC.

**References**

1. Roy B., Friesen W.J., Tomizawa Y., Leszyk J.D., Zhuo, J., Johnson B., Dakka, J., Trotta C.R., Xue X.; Mutyam V.; Keeling K.M.; Mobley J.A.; Rowe S.M.; Bedwell D.M., Welch E.M., Jacobson A. Ataluren stimulates ribosomal selection of near-cognate tRNAs to promote nonsense suppression. *PNAS.* 2016, *113*, 12508-12513.

2. Lentini L, Melfi R, Di Leonardo A, Spinello A, Barone G, Pace A, Palumbo Piccionello A and Pibiri I. Towards a rationale for the PTC124 (Ataluren) promoted read through of premature stop codons: a computational approach and GFP-reporter cell-based assay, Mol. Pharm. 11, 2014, 653–664.

3. Pibiri I, Lentini L, Tutone M, Melfi R, Pace A and Di Leonardo A. Exploring the readthrough of nonsense mutations by non-acidic Ataluren analogues selected by ligand- based virtual screening, Eur. J. Med. Chem. 2016, 122, 429–435.

4. Pibiri I, Lentini L, Melfi R, Tutone M, Baldassano S, Ricco Galluzzo P, DiLeonardo A, Pace A. Rescuing the CFTR protein function: Introducing 1,3,4-oxadiazoles as translational readthrough inducing drugs-European Journal of Medicinal chemistry 2018, 159, pp.126-142.

**Acknowledgment**. FFC#3/2017 funded by FFC and supported by Delegazione FFC di Palermo, Delegazione FFC di Vittoria, Ragusa e Siracusa, Delegazione FFC di Catania Mascalucia

## NON TUBERCOLOUS MYCOBACTERIA

### O15 Preclinical evaluation of liposomes carrying bioactive lipids as an immune therapeutic tool against in vivo infection with *Mycobacterium abscessus*

#### Riva C^1^, Poerio N^2^, Rossi M^1^, Gona F^1^, Tortoli E^1^, Fraziano M^2^, Cirillo DM^1^

##### ^1^Emerging Bacterial Pathogens Unit, IRCCS San Raffaele Scientific Institute, Milano, Italy; ^2^ Department of Biology, University of Rome Tor Vergata, Rome, Italy

###### **Correspondence:** Cirillo DM (cirillo.daniela@hsr.it)

**Background and rationale**

*M. abscessus* (MA) is an emerging multidrug resistant (MDR) non-tuberculous mycobacteria (NTM) that affects cystic fibrosis (CF) patients, and is often associated with a dramatic decline in lung function [1]. Liposomes carrying bioactive lipids represent a new pharmacologic approach that are able to enhance bactericidal innate immunity, against multidrug resistant (MDR) pulmonary infections [2,3].

**Hypothesis and objectives**

The main goal of the present study was to evaluate the effect of different apoptotic body-like liposomes (ABLs) on *in vitro* and *in vivo* MA infection. The MA infection *in vitro* allowed us to investigate the mechanism of action of ABLs on macrophage phagocytosis mechanism.

**Essential methods**

We set up different *in vitro* infections with MA reference strain (ATCC 19977) on human pro-monocytic THP-1 leukemia cell line (dTHP-1) to investigate the effect of ABLs on phagocytic mechanism. We validated the effect of different ABLs (ABL/PA, ABL/PI3P, ABL/PI5P, ABL/AA, ABL/LBPA and ABL/S1P) *in vitro* with dTHP-1 cells and in C57Bl/6 mice chronically infected with MA. At different time points, mice lungs, liver and spleen were processed for microbiological analysis. Inflammatory response and histological analysis were evaluated in total lungs.

**Results**

*In vitro* results showed that ABL/PA, ABL/PI3P and ABL/PI5P were more effective to increase the phagocytic and the intracellular microbicidal activity of human dTHP1 infected with MA than ABL/AA, ABL/LBPA and ABL/S1P. Then we established a consistent pulmonary infection in immunocompetent mice up to 36 days with MA and at different time point mice were treated with the best 3 ABLs (ABL/PA, ABL/PI3P and ABL/PI5P) selected in *in vitro* experiments. Results showed that, despite the absence of effect in granuloma reduction at parenchymal level, ABLs treatment statistically reduced both lung’s bacterial burden and inflammatory response.

**Conclusions**

ABLs could represent a novel immunotherapeutic strategy to treat pulmonary infection by drug-resistant MA in CF patients.

**References**

1. de Vrankrijker AM, Wolfs TF, van der Ent CK. Challenging and emerging pathogens in cystic fibrosis. Paediatr Respir Rev. 2010 Dec;11(4):246-54. doi: 10.1016/j.prrv.2010.07.003. 2. Del Porto P1, Cifani N, Guarnieri S, Di Domenico EG, Mariggiò MA, Spadaro F, Guglietta S, Anile M, Venuta F, Quattrucci S, Ascenzioni F. Dysfunctional CFTR alters the bactericidal activity of human macrophages against Pseudomonas aeruginosa. PLoS One. 2011;6(5):e19970. doi: 10.1371/journal.pone.0019970.

3. Greco E, Quintiliani G, Santucci MB, Serafino A, Ciccaglione AR, Marcantonio C, Papi M, Maulucci G, Delogu G, Martino A, Goletti D, Sarmati L, Andreoni M, Altieri A, Alma M, Caccamo N, Di Liberto D, De Spirito M, Savage ND, Nisini R, Dieli F, Ottenhoff TH, Fraziano M. Janus-faced liposomes enhance antimicrobial innate immune response in Mycobacterium tuberculosis infection. Proc Natl Acad Sci USA. 2012 May 22;109(21):E1360-8. doi: 10.1073/pnas.1200484109.

**Acknowledgment**. FFC#16/2018 funded by FFC and supported by Delegazione FFC di Vittoria Ragusa e Siracusa and Delegazione FFC di Catania Mascalucia

### O16 Induction of viable but non-culturable forms, possibly responsible for treatment failure, in “in vitro” biofilms of *Pseudomonas aeruginosa*. Role of antibiotics and antibiotic concentrations

#### Biavasco F, Mangiaterra G, Cedraro N, Vaiasicca S, Bizzaro D, Vignaroli C

##### Department of Life and Environmental Sciences, Polytechnic University of Marche, Ancona

###### **Correspondence:** Biavasco F (f.biavasco@univpm.it)

**Background and rationale**

Persistent bacteria, including the Viable But Non-Culturable (VBNC) forms, are involved in the recurrent and chronic *Pseudomonas aeruginosa* (PA) lung infections affecting Cystic Fibrosis (CF) patients [1]. VBNC development can be induced by a variety of stressors [2], such as exposure to toxic compounds (e.g. antibiotics) and nutrient depletion, that are typically found in the CF lung.

**Hypothesis and objectives.** We have previously demonstrated the involvement of tobramycin (T) in the abundance and stability of VBNC PA forms in *in vitro* biofilms starved for 5 months. This year we set out to gain further insights into i) the role of T and of different T resistance mechanisms and ii) the involvement of additional stressors found in the CF lung in the abundance of VBNC cells in PA biofilms.

**Essential methods**

Three PA strains characterized by different T resistance mechanisms – two clinical CF strains (PA C30, showing *mexXY-*based low-level resistance and PA AR86, showing *ant(2”)*-*Ia*-based high-level resistance) and the PAO1 reference strain – were used in the study. We exposed 48h *in vitro* biofilms to different stress conditions: maintenance in NN broth (NN); high salt concentrations; catabolite accumulation; each unsupplemented or supplemented with subinhibitory T concentrations. Biofilms were tested for 5 months (PA30 and PA AR86) or 7 days (PAO1) for VBNC cell content using culture methods, qPCR [3] and flow cytometry after live/dead staining.

**Results**

After 5-month exposure to NN+T, VBNC abundance was greater in PA C30 (95% of all live cells) than in PA AR86 biofilms (50%), which indicates that there were 50% culturable persisters in the latter biofilms. NN exposure induced similar effects on biofilms of both strains (90% of VBNC cells). The PAO1 biofilms, tested after 7-day exposure, showed a greater VBNC abundance in response to high salt concentrations (90%) and catabolite accumulation (99%) than to NN (only culturable PA cells).

**Conclusions**

These findings demonstrate: i) a variable and strain-specific effect of T in inducing the VBNC phenotype after long-term exposure; ii) its involvement in the development of VBNC or persistent culturable forms in presence of typical CF-related stress conditions; and iii) the need for a culture-independent microbiological diagnosis to properly monitor PA colonization of CF lung.

**References**

1. Deschaght P, Schelstraete P, Van Simaey L, Vanderkercken M, Raman A, Mahieu L, Van Daele S, De Baets F, Vaneechoutte M. Is the improvement of CF patients, hospitalized for pulmonary exacerbation, correlated to a decrease in bacterial load? PLoS One. 2013 Nov 29;8(11):e79010. doi: 10.1371/journal.pone.0079010. eCollection 2013.

2. Li L, Mendis N, Trigui H, Oliver JD, Faucher SP. The importance of the viable but non-culturable state in human bacterial pathogens. Front Microbiol. 2014 Jun 2;5:258. doi: 10.3389/fmicb.2014.00258. eCollection 2014.

**3.** Mangiaterra G, Amiri M, Di Cesare A, Pasquaroli S, Manso E, Cirilli N, Citterio B, Vignaroli C, Biavasco F. Detection of viable but non-culturable Pseudomonas aeruginosa in cystic fibrosis by qPCR: a validation study. BMC Infect Dis. 2018 Dec 27;18(1):701. doi: 10.1186/s12879-018-3612-9.

**Acknowledgment** FFC#13/2017 funded by FFC and supported by LIFC Toscana onlus

## CF INFLAMMATION: THERAPEUTICAL APPROACHES?

### O17 Thymosin alpha 1 in cystic fibrosis: from the lung to the gut

#### Pariano M, Renga G, Stincardini C, Borghi M, Sellitto F, Sforna L, D’Onofrio F, Costantini C, Romani L and Bellet MM

##### Dipartimento di Medicina Sperimentale, Università degli Studi di Perugia, Italy

###### **Correspondence:** Romani L (marina_bellet@yahoo.com)

**Background and rationale**

The gastrointestinal system is among the earliest organs affected in cystic fibrosis (CF) (1, 2). Gut manifestations includes, among all, mucus accumulation, recurrent infections and chronic inflammation. The aim of this project is centered around the effects on CF of thymosin alpha1 (Tα1), a naturally occurring polypeptide used worldwide as an immunomodulatory with an excellent safety profile (3). By acting on autophagy and cellular proteostasis, Tα1 displayed multiple combined properties that may oppose CF symptomatology in the lung: reduce inflammation and, possibly, improve CFTR function (4). We previously demonstrated that Tα1 in the lung reduces inflammation by activating the tolerogenic pathway of tryptophan catabolism via the immunoregulatory enzyme indoleamine 2,3-dioxygenase (IDO1) (4).

**Hypothesis and objectives**

The present project aimed at extending our previous results obtained in the lung to the intestine and pancreas, to provide a more complete picture of the multiple effects of Tα1 in CF. The hypothesis was that Tα1 may regulate inflammation and autophagy via IDO1 in the intestinal tract in CF. The objectives of the project have been the evaluation of the ability of Tα1 to regulate inflammation and antimicrobial resistance in the gut, as well as pancreas functionality in CF, and to restore the IDO1-autophagy pathway in these tissues.

**Essential methods**

The effect of both Tα1 and the pharmaceutical formulation ZADAXIN were evaluated in different murine experimental models:

1. Model of high fat diet-induced intestinal inflammation, disbiosis and pancreatic dysfunction, to define the efficacy of Tα1 in a model of “leaky gut”
2. Model of gastrointestinal infection with *C. albicans* in *Cftr*^*F508del*^ mice and Ido1^-/-^ mice, to evaluate the effect of Tα1 and ZADAXIN in an inflamed gut, and its dependency on IDO1 activation

**Results**

The results of our project have indicated that Tα1, as well as ZADAXIN, is able to ameliorate inflammation and promote barrier function in the gut of *Cftr*^*F508del*^, and this effect appears to be IDO1-dependent.

**Conclusions**

Studies are underway to better define the activity of Tα1 *in vivo*, its mechanism of action, and to provide further evidence supporting its ability to modulate CFTR function. These studies will provide the foundation for repurposing a drug approved for other indications for the treatment of CF.

**References**

1. Ooi, C.Y. and Durie P.R., Cystic fibrosis from the gastroenterologist's perspective. Nat Rev Gastroenterol Hepatol, 2016. 13(3): p. 175-85.

2. Sathe, M.N. and Freeman A.J., Gastrointestinal, Pancreatic, and Hepatobiliary Manifestations of Cystic Fibrosis*.* Pediatr Clin North Am, 2016. 63(4): p. 679-98.

3. Goldstein, A.L. and Goldstein A.L., From lab to bedside: emerging clinical applications of thymosin alpha 1*.* Expert Opin Biol Ther, 2009. 9(5): p. 593-608.

4. Romani, L. et al., Thymosin α1 represents a potential potent single-molecule-based therapy for cystic fibrosis. Nat Med, 2017. 23(5): p. 590-600

**Acknowledgment** FFC#21/2018 funded by FFC and supported by Latteria Montello, Delegazione FFC di San Giuseppe Vesuviano, Con Cecilia amici della ricerca

### O18 Preclinical testing in cystic fibrosis of a repurposed molecule targeting HMGB1

#### De Leo F^1,2^ , Rossi A^3^, Cigana C^3^ , Ranucci S^3^, De Fino I^3^, Musco G^1^, Bragonzi A^3^ and Bianchi ME^2,4^

##### ^1^Biomolecular NMR Unit, IRCCS San Raffaele Scientific Institute; ^2^Università Vita-Salute San Raffaele, Milan, Italy; ^3^Infections and Cystic Fibrosis Unit, Division of Immunology, Transplantation and Infectious Diseases San Raffaele Scientific Institute; ^4^Chromatin dynamics unit, IRCCS San Raffaele Scientific Institute, via Olgettina 58, 20132 Milan, Italy

###### **Correspondence:** Bianchi ME (bianchi.marco@hsr.it)

**Background and rational**

High-mobility group box 1 protein is a Damage-associated molecular pattern (DAMP) protein that promotes and sustains inflammation. It is elevated in CF sputum and was reported as CF biomarker. Monoclonal antibodies (mAb) blocking HMGB1 significantly protect against *P. aeruginosa* infection, neutrophil recruitment and lung injury [1]. However, mAb treatment is expensive and infeasible. We recently identified a small molecule, pamoic acid (PAM), that efficiently inhibits HMGB1 activity in cellular models. Furthermore, it is known that PAM cannot cross lipid membranes, so that it might be particularly suitable for direct aerosol delivery in the lung.

**Hypothesis and objectives**

The objective of this project was proving that aerosol delivery of PAM can ameliorate neutrophilic inflammation and lung damage in C57 mice infected with *P. aeruginosa*.

**Essential methods**

PAM was tested in preclinical murine models of acute [2,3] and chronic [4,5] *P. aeruginosa* respiratory infection. Specifically, we performed:

1. Toxicity experiments to explore the range of doses to be used;
2. PAM efficacy experiments in acute respiratory infection;
3. PAM identification and quantification by spectroscopic techniques;
4. PAM efficacy experiments in chronic respiratory infection.

**Results**

PAM does not have acute toxicity when delivered by aerosol at concentrations ≤3 mM. PAM at 3 mM concentration shows significant efficacy in reducing inflammatory cells in the bronco-alveolar lavage in an acute model of *Pseudomonas* infection, and may be effective in a chronic model even in a lower concentration range.

Of note, we focused on the chemical properties of PAM, which suggested an advantage in direct lung delivery via aerosol, to avoid systemic adsorption. However, we cannot exclude that formulations that allow systemic diffusion might be more effective than aerosol. PAM might be prematurely cleared; in fact, we still do not know the local (tissue and BAL) concentration of PAM, which is relevant to interpret the efficacy.

**Conclusions**

Up to date PAM is the first drug candidate to display efficacy against inflammation in a mouse model of chronic respiratory infection that reproduces CF lung disease [4]. Further investigation is needed, but we consider the present results as positive and promising.

**References**

1. Entezari M, Weiss DJ, Sitapara R, Whittaker L, Wargo MJ, Li J, Wang H, Yang H, Sharma L, Phan BD, Javdan M, Chavan SS, Miller EJ, Tracey KJ, Mantell LL Inhibition of High-Mobility Group Box 1 Protein (HMGB1) Enhances Bacterial Clearance and Protects against Pseudomonas Aeruginosa Pneumonia in Cystic Fibrosis. *Mol. Med.* **2012**.

2. Bragonzi, A. Murine Models of Acute and Chronic Lung Infection with Cystic Fibrosis Pathogens. *International Journal of Medical Microbiology*. 2010.

3. Facchini M, De Fino I, Riva C, Bragonzi A. Long Term Chronic Pseudomonas Aeruginosa Airway Infection in Mice. *J. Vis. Exp.* **2014**, No. 85.

4. Cigana C, Lorè NI, Riva C, De Fino I, Spagnuolo L, Sipione B, Rossi G, Nonis A, Cabrini G, Bragonzi, A. Tracking the Immunopathological Response to Pseudomonas Aeruginosa during Respiratory Infections. *Sci. Rep.* **2016**, *6* (1), 21465.

5. Cigana C, Bernardini F, Facchini M, Alcalá-Franco B, Riva C, De Fino I, Rossi A, Ranucci S, Misson P, Chevalier E, Brodmann M, Schmitt M, Wach A, Dale GE, Obrecht D, Bragonzi A. Efficacy of the Novel Antibiotic POL7001 in Preclinical Models of Pseudomonas Aeruginosa Pneumonia. *Antimicrob. Agents Chemother.* **2016**, *60* (8), 4991–5000.

**Acknowledgment** FFC#22/2018 funded by FFC and supported by Delegazione FFC di Pesaro

### O19 Evaluation of anti-inflammatory treatments for CF lung disease in murine models of lung infection in vivo

#### Dechecchi MC^1^, Guaragna A^2^

##### ^1^Laboratory of Molecular Pathology, University Hospital of Verona- Italy; ^2^Department of Chemical Sciences, University of Napoli Federico II- Napoli, Italy

###### **Correspondence:** Dechecchi MC (mcristina.dechecchi@gmail.com)

**Background and rationale**

Despite exciting developments, CFTR restoration for all CF people is challenging and may not be sufficiently efficacious in patients with irreversible lung damage. As inflammation contributes to lung damage, research is aimed at finding new anti-inflammatory drugs, to replace corticosteroids or ibuprofen, which possess many well-known and important side effects in addition to the great predicted benefits [1]. Thanks to previous FFC grants we described two promising molecules: beta-sitosterol (BSS) [2] and miglustat [3] that have been widely tested for efficacy and safety in clinical pharmacology, thus stimulating us to a repurposing strategy toward CF lung inflammation.

**Hypothesis and objectives**

BSS has anti-inflammatory activity in CF bronchial cells and l enantiomer of miglustat produces an anti-inflammatory effect in mice acutely infected by *P.aeruginosa* without increasing the bacterial load or inducing toxicity [4]. Importantly l-miglustat does not inhibit alpha-glucosidase [5] thus excluding the undesired side effects on intestinal absorption and diarrhea observed with d-enantiomer of miglustat.

This pilot project analysed BSS and l miglustat in relevant murine models of lung infection.

**Essential methods**

BSS is commercially available whereas l-miglustat was synthesized and purified by A. Guaragna (Department of Chemical Sciences, University of Napoli Federico II- Napoli, Italy). BSS and l-miglustat were evaluated in relevant models of airway infection for their effect in reducing the inflammatory response to *P.aeruginosa*. Wild type mice were treated with BSS or l- miglustat by gavage before infection with *P.aeruginosa* and their effect on inflammatory response was tested in terms of: *i*) safety; *ii*) evaluation of lung inflammation; *iii*) ability of mice to clear bacteria.

**Results**

We found a dose dependent reduction of bacteria recovered in broncholavage (BAL) and lungs of mice infected by *P.aeruginosa,* after treatment with BSS*.* A decrease of neutrophils and increase of alveolar macrophages recruited in BAL accompanied by an overall improvement of health parameters in BSS treated mice were also found. Surprisingly, l-miglustat showed anti-infective activity in chronically infected mice, reduced neutrophils and increased bacterial clearance.

**Conclusions**

Results from our pre-clinical investigation in relevant models of airway infection could provide a proof of concept for planning clinical trials. BSS has been already tested in clinical trials as adjuvant to statins in dyslipidemia. It could be repositioned as anti- inflammatory drug for CF lung disease. l-miglustat could obtain an orphan drug designation as anti-inflammatory/anti-infective agent against chronic *P. aeruginosa* infections.

**References**

1. Bell SC, Mall MA, Gutierrez H et al. The future of cystic fibrosis care: a global perspective. Lancet Respir Med. 2019: S2213-2600(19)30337-6.

2. Lampronti I, Dechecchi MC, Rimessi A et al. β-Sitosterol Reduces the Expression of Chemotactic Cytokine Genes in Cystic Fibrosis Bronchial Epithelial Cells. Front Pharmacol. 2017 May 12;8: 236.

3. Dechecchi MC, Nicolis E, Mazzi P et al. Modulators of sphingolipid metabolism reduce lung inflammation. Am J Respir Cell Mol Biol. 2011Oct;45(4):825-33.

4. De Fenza M, D'Alonzo D, Esposito A et al. Exploring the effect of chirality on the therapeutic potential of N-alkyl-deoxyiminosugars: anti-inflammatory response to Pseudomonas aeruginosa infections for application in CF lung disease. Eur J Med Chem. 2019 Aug 1;175:63-71.

**Acknowledgment**. FFC#23/2018 funded by FFC and supported by Emanuela Cricri e amici della ricerca, Delegazione FFC di Villa D'Almè – Bergamo, Gruppo di Sostegno FFC di Magenta, Donazione privata

### O20 Enabling pulmonary delivery of siRNA in cystic fibrosis lung inflammation: therapeutic potential of hybrid lipid/polymer nanoparticles

#### Ivana d’Angelo^1^, Gabriella Costabile^2^, Gemma Conte^1,3^, Domizia Baldassi^2^, Emma Mitidieri^3^, Roberta d’Emmanuele di Villa Bianca^3^, Fabiana Quaglia^3^, Paola Brocca^4^, Ersilia Fiscarelli^5^, Raffaella Sorrentino^3^, Olivia. M. Merkel^2^, Francesca Ungaro^3^

##### ^1^Di.S.T.A.Bi.F., Università della Campania "Luigi Vanvitelli", Caserta, Italy; ^2^Dept. of Pharmacy, Pharmaceutical Technology and Biopharmacy, Ludwig-Maximilians-Universität München, München, Germany; ^3^Dip. di Farmacia, Università degli Studi di Napoli “Federico II”, Napoli, Italy; ^4^Dip di Biotecnologie Mediche e Medicina Traslazionale, Università degli Studi di Milano, Milano, Italy; ^5^Laboratorio di Microbiologia FC, Ospedale Pediatrico Bambino Gesù, Roma, Italy

###### **Correspondence:** Francesca Ungaro (ungaro@unina.it)

**Background and rationale**

The down-regulation of genes directly involved in the pathogenesis of severe lung diseases through pulmonary delivery of short RNA fragments, also known as siRNA, has gained recently remarkable research interest, especially in cystic fibrosis (CF) [1]. Nevertheless, the unsuccessful history of inhaled siRNA points out the urgent need of an appropriate formulation strategy to move them from the laboratory to the bedside [2].

**Hypothesis and objectives**

The generation of breakthrough technologies and their translation into new pharmaceutical products is crucial for CF treatment. During previous FFC#23/2017 project, the most adequate formulation conditions to produce inhalable hybrid nanoparticles (hNPs) made up of a combination of lipids and polymers for siRNA delivery were identified. The developed hNPs displayed optimal aerosolization properties, were stable in simulated mucus and efficiently entrapped a siRNA pool against one of the most critical signals in evoking the inflammatory response in CF, the nuclear factor-κB (NF-κB). The aim of the present project is to go in depth into the in vitro/in vivo therapeutic potential of optimised hNPs.

**Essential methods**

hNPs delivering a siRNA pool against NF-κB were prepared from biodegradable polymers and biocompatible phospholipids [3]. The behaviour of hNPs upon contact with simulated mucus and human sputum from CF was evaluated through a combination of techniques. Uptake and efficacy of siRNA-loaded hNPs were evaluated in different human airway cell culture models, providing a tool to optimise hNP properties for in vivo pulmonary delivery. In vivo studies were performed in rats challenged intratracheally with LPS from E. Coli to induce pulmonary inflammation.

**Results**

In vitro studies demonstrated that the developed siRNA-loaded hNPs may penetrate lung extracellular barriers, as CF mucus, allowing a significantly higher uptake in human bronchial epithelial cells as compared to both free siRNA and siRNA/lipofectamine complexes. As a result, significant NF-κB downregulation up to 72 h was observed in human bronchial epithelial cells treated with optimised siRNA-loaded hNPs. Finally, preliminary efficacy studies upon intratracheal administration in LPS-challenged rats highlighted the potential of the developed siRNA-loaded to downregulate NF-κB also in vivo.

**Conclusions**

The correct operating conditions to produce nanoparticles for prolonged release of siRNA in CF lung have been identified. Optimized nanoparticles can move to further *in vivo* preclinical studies, which are essential to translate the technology under development from labs to the clinics. The development of a siRNA delivery system already engineered for in vivo inhalation and transfection might shorten the time to translation to patients, providing a therapeutic platform to address multiple targets that are still considered “undruggable” in CF.

**References**

1. Setten RL, Rossi JJ & Han S. The current state and future directions of RNAi-based therapeutics. Nat Rev Drug Discovery. 2019; 18, 421-446.

2. Thanki K, Blum KG, Thakur A, Rose F, Foged C. Formulation of RNA interference-based drugs for pulmonary delivery: challenges and opportunities. Ther. Deliv. 2018; 9(10), 731–749.

3. d’Angelo I, Costabile G, Durantie E, Brocca P, Rondelli V, Russo A, Russo G, Miro A, Quaglia F, Petri-Fink A, Rothen-Rutishauser B, Ungaro F. Hybrid Lipid/Polymer Nanoparticles for Pulmonary Delivery of siRNA: Development and Fate Upon In Vitro Deposition on the Human Epithelial Airway Barrier. J Aerosol Med Pulm Drug Deliv. 2018; 31(3): 170- 181.

**Acknowledgement** FFC#25/2018 funded by FFC and supported by Delegazione FFC di Montebelluna.

### O21 Properties of airway mucus in cystic fibrosis: their modification by changes in the activity of CFTR and after application of bicarbonate

#### Ferrera L, Gianotti A, Delpiano L, Capurro V

##### U.O.C. Genetica Medica, Istituto Giannina Gaslini, Genova, Italy

###### **Correspondence:** Ferrera L (loretta.ferrera@ge.ibf.cnr.it)

**Background and rationale**

Most of the problems of patients affected by cystic fibrosis (CF) are due to the defective mucociliary cleareance caused by a very thick mucus. We demonstrated that the viscoelastic properties of CF mucus improve with the direct application of lumacaftor. Thus, we hypothesised that the rescue of the F508del-CFTR function by lumacaftor could increase the bicarbonate secretion, recovering the correct airway surface liquid (ASL) and pH homeostasis. According to our hypothesis, a direct treatment of airways with bicarbonate, determining an increase of the ASL pH, would result beneficial in the viscoelastic properties of the mucus.

**Hypothesis and objectives**

The aims of the project were: 1) to investigate whether the bicarbonate treatment modify the water reabsortion in the ASL; 2) to verify the role of the bicarbonate direct treatment on the pH of the ASL; 3) to study the role of the airway bicarbonate on the viscoelastic properties of the ASL mucus to examine whether the mutant CFTR rescue by lumacaftor is correlated with the bicarbonate transport and pH modifications in the ASL.

**Essential methods**

We used primary bronchial cells monolayers from normal subjects and CF patients as epithelial models. Bicarbonate was directly applied to the apical surface of the monolayers. After 48 hours, water reabsorption, pH and mucus microviscosity, measured by the displacement of fluorescent nanobeads, characterises the properties of the ASL.

**Results**

Our results clearly indicate that the treatment with bicarbonate determines increase of ASL and pH, and significantly reduces the mucus viscosity in treated CF epithelia. Data obtained in this work will be useful for the design strategies for CF treatment patients using inhaled bicarbonate, and improve the protocols that are been used in a clinical trial. Noteworthy, bicarbonate might represent a mutation-independent and low cost therapy to clear out the mucus that accumulates in the airways, reducing the risk of infections, and improve lung function.

**Acknowledgment** FFC#12/2016 funded by FFC and supported by Delegazione FFC di Genova, Delegazione FFC di Pomezia Roma, Delegazione FFC di Massafra con Delegazione FFC di Taranto e Gruppo di Sostegno FFC di Alberobello

### O22 Testing the anti-inflammatory effects of matrix metalloprotease inhibitors in P. aeruginosainfected CFTR-knockout mice by in vivo imaging techniques

#### Sandri A^1^, Lleò MM^1^, Boschi F^2^

##### ^1^Department of Diagnostics and Public Health, University of Verona, Italy; ^2^Department of Computer Science, University of Verona, Italy

###### **Correspondence:** Boschi F (federico.boschi@univr.it)

(FFC#21/2017)

**Background and rationale** P. aeruginosa secreted proteases interfere with key host immune processes and degrade lung tissue [1]. Thus, molecules interfering with bacterial proteases might limit host inflammatory response and lung damage. Modern in vivo imaging tools can allow to assess the anti-inflammatory effects of these molecules in vivo.

**Hypothesis and objectives** We aimed at setting-up a convenient, non-invasive, in vivo imaging model to monitor lung inflammation in CF mice with P. aeruginosa acute lung infection, and to evaluate the possible anti-inflammatory effects of molecules interfering with proteases, such as protease inhibitors Marimastat and Ilomastat.

**Essential methods** P. aeruginosa acute lung infection was established by intratracheal instillation in wildtype (WT) and CFTR-knockout (KO) C57BL/6 transgenic mice expressing the luciferase gene under control of bovine IL-8 promoter [2]. Transgenic mice were treated with protease inhibitors Marimastat and Ilomastat, and lung inflammation was monitored by in vivo bioluminescence imaging. In vitro, effects of protease inhibitors on P. aeruginosa growth and viability were evaluated.

**Results** Acute lung infection with P. aeruginosa PAO1 strain was established in both WT and KO mice. The infection induced IL-8-dependent bioluminescence emission indicating lung inflammation, along with low mortality of the animals in the first 48 hours. In infected mice with ongoing inflammation, intratracheal treatment with 150μM Marimastat and Ilomastat reduced the bioluminescence signal in comparison to untreated, infected animals. Bacterial load in the lungs was not affected by the treatment, while in vitro the same dose of Ilomastat and Marimastat did not affect P. aeruginosa growth and viability. No adverse effects due to treatment with protease inhibitors were observed in mice.

**Conclusions** Our results show that protease inhibition elicits beneficial effects in mice by reducing the lung inflammation caused by P. aeruginosa infection. Thus, Ilomastat and Marimastat might be potential candidate molecules for the treatment of patients with P. aeruginosa infection, encouraging further studies on protease inhibitors and their possible application in cystic fibrosis. Particularly, inhalable formulations [3] could be a preferential therapy for CF patients, allowing local airways treatment.

**References**

1. Kipnis E, Sawa T, Wiener-Kronish J. Targeting mechanisms of Pseudomonas aeruginosa pathogenesis. Med Mal Infect. 2006; 36(2):78-91.

2. Bergamini G, Stellari F, Sandri A, M Lleo M, Donofrio G, Ruscitti F, Boschi F, Sbarbati A, Villetti G, Melotti P, Sorio C. An IL-8 transiently transgenized mouse model for the in vivo long-term monitoring of inflammatory responses. J Vis Exp. 2017; 125:e55499.

3. Pemberton PA, Cantwell JS, Kim KM, Sundin DJ, Kobayashi D, Fink JB, Shapiro SD, Barr PJ. An inhaled matrix metalloprotease inhibitor prevents cigarette smoke-induced emphysema in the mouse. COPD. 2005; 2(3):303-10.

**Acknowledgment**. FFC#21/2017 funded by FFC and supported by Delegazione FFC di Boschi Sant'Anna Minerbe "Alla fine esce sempre il sole”, Delegazione FFC di Massafra

